# User experience and care for older people transitioning from hospital to home: Patients’ and carers’ perspectives

**DOI:** 10.1111/hex.12646

**Published:** 2017-11-09

**Authors:** Jacqueline Allen, Alison M. Hutchinson, Rhonda Brown, Patricia M. Livingston

**Affiliations:** ^1^ School of Nursing and Midwifery Deakin University Geelong Burwood Vic. Australia; ^2^ Centre for Quality and Patient Safety Monash Health Partnership Monash Health Burwood Vic. Australia; ^3^ Faculty of Health & School of Nursing and Midwifery Deakin University Geelong Burwood Vic. Australia

**Keywords:** discharge planning, older people, qualitative study, transitional care

## Abstract

**Background:**

Transitioning from hospital to home is challenging for many older people living with chronic health conditions. Transitional care facilitates safe and timely transfer of patients between levels of care and across care settings and includes communication between practitioners, assessment and planning, preparation, medication reconciliation, follow‐up care and self‐management education. To date, there is limited understanding of how to actively involve care recipient service users in transitional care.

**Objective:**

This study was part of a larger research project. The objective of this article was to report the first study phase, in which we aimed to describe user experience pertaining to patients and carers.

**Design, setting and participants:**

The study design was qualitative descriptive using interviews. Patients (n = 19) and carers (n = 7) participated in semi‐structured interviews about their experience of transition from hospital to home in an urban Australian health‐care setting. Interview data were analysed using thematic analysis.

**Findings:**

All participants reported that they needed to become independent in transition. Participants perceived a range of social processes supported their independence at home: supportive relationships with carers, caring relationships with health‐care practitioners, seeking information, discussing and negotiating the transitional care plan and learning to self‐care.

**Discussion:**

Findings contribute to our understanding that quality transitional care should focus on patients’ need to regain independence. Social processes supporting the capacities of patients and carers should be emphasized in future initiatives.

**Conclusion:**

Future transitional care interventions should emphasize strategies to enable negotiation for suitable supports and assist care recipients to overcome barriers identified in this study.

## 1 INTRODUCTION

Transitioning from hospital to home is challenging for many older people living with chronic health conditions and for their families. Transitional care interventions facilitate safe and timely transfer of patients between levels of care and across care settings.^1^ Suboptimal transitional care is problematic in human and health service efficiency terms because it results in unmet needs at home, unnecessary early readmission to hospital, and unwanted permanent placement in residential care.^2^, ^3^, ^4^ Researchers have attributed ineffective transitional care to long‐standing difficulties in care integration because of inadequate inclusion of older people and their carers in transitional care.^5^, ^6^, ^7^, ^8^ Care integration is further complicated by complex contexts of health care affecting service provision and characterized by service fragmentation and by changing patterns of demand on health services related to the ageing population.^9^, ^10^, ^11^ This study was part of a larger research project. The objective of this article was to report the first study phase, in which we aimed to describe user experience pertaining to patients and carers. In this first study phase, we present findings from semi‐structured interviews with patients and carers.

### 1.1 Transitional care

The goal of optimal transitional care is to promote safe and timely transfer of patients between levels of care and across care settings.^1^, ^12^, ^13^ Transitional care has been studied within disease‐focused models of care^10^ and it has been studied as an intervention in aged care.^14^ Transitional care is not strictly defined by beginning and end points; it includes pre‐hospital discharge considerations and immediate post‐hospital discharge follow‐up at the next location of care.^12^, ^15^ Transitional care can be considered a part of integrated care, where multiple services provide home‐based care over long periods of time^16^ and it can be considered a part of prevention of readmission programs within long‐term chronic disease management initiatives.^17^ Although transitional care is related to integrated care and prevention of readmission programs, it is considered a conceptually distinct category of care interventions.^15^ According to Coleman and Boult,^1^ there are a number of essential elements and processes in quality transitional care: communication between practitioners about the discharge assessment and plan of care; preparation of the person and carer for the care transition; reconciliation of medications at transition; a plan for follow‐up care; and patient education about self‐management.

Some interventions emphasizing preparation of patients and carers for discharge have been evaluated using pre/post‐designs and found improved quality of discharge care for older people.^18^ Two well‐researched transitional care interventions in the USA, the Care Transitions Intervention,^1^, ^19^, ^20^ and the Transitional Care Model^21^, ^22^, ^23^, ^24^, ^25^ have been developed, implemented and tested using randomized controlled trial designs. Each of these interventions emphasized care integration in addition to transitional care assessment and planning, medication reconciliation, preparation and involvement of the older person and carer, and self‐management support. Each intervention involves a health‐care practitioner in a specified role to support transitional care. In the Care Transitions Intervention, a transition coach is required,^19^ and in the Transitional Care Model, an advanced practice nurse is required.^23^, ^25^ Coleman and colleagues, and Naylor and associates have demonstrated significant reductions in re‐admission rates to hospital, and high patient satisfaction with care following their respective interventions.

Despite this research,^19^, ^23^, ^25^ the involvement of older people and carers in decisions about their care transitions remains problematic in practice.^5^, ^26^, ^27^, ^28^, ^29^ This is due to a range of barriers including limited support for patients and carers to engage in quality discussions about their care needs with health‐care practitioners, the fragmentation of services and need for fast throughput.^5^, ^30^ There is limited understanding of how to involve patient and carer service users in service design, and how to employ the “user experience” of older people and their carers/families in the development of transitional care interventions within systems characterized by acute, subacute and community‐based care.^28^, ^31^ Moreover, detailed consideration of the influences of local contextual factors, including enabling and constraining factors, on users’ experience could provide important insights for consideration in new transitional care initiatives. This study aimed to address these issues in service delivery and expand on the evolving knowledge base in relation to transitional care.

### 1.2 Research question

How do older people and their carers/families as care recipient service users, experience discharge and transitional care across the trajectories of acute, subacute and community care?

## 2 METHODOLOGY

### 2.1 Study design

The study design was qualitative exploratory descriptive using interviews.

### 2.2 User experience as a social process

The framework adopted for the larger study is located broadly within constructivism, understood as social processes simultaneously creative of and created by people through interactions with their social context, and with each other.^32^, ^33^ User experience refers to how the user of a health service feels and thinks about the service while they are using it.^34^ The social world, particular to the health‐care environment, shapes user's interpretations of the meaning of their experiences of health care, and users’ interpretations of the meaning of their experiences of health care can in turn shape the social world of the relevant health‐care environment. Hence, user experience in health‐care contexts, including transitional care, is itself a social process. The social process of user experience in transitional care formed the principal meaning unit for qualitative data analysis.

### 2.3 Setting

A large metropolitan public health‐care network in the Australian city of Melbourne formed the study setting. Patients and carers were recruited from two inpatient sites including a ward providing acute general medicine services, and a subacute ward providing rehabilitation services for older people. These wards and programs were selected because they provide different care to older people, acute approaches to care in acute medicine, and chronic care and rehabilitation. Additionally, patients were discharged to the care of their general practitioner (GP) and community‐based care including community nursing, housekeeping and personal care services. This maximized variation and opportunities for information about different user experiences in different inpatient settings and with continuing care in the community.

### 2.4 Ethics approvals

Following the guidelines of Australia's National Health and Medical Research Council,^35^ ethics approvals were obtained from the Human Research Ethics Committees at the participating health‐care network and Deakin University. Patients and carers participated voluntarily in the study. Participants provided verbal and written consent following an explanation of the study guided by the Participant Information Form. Data were de‐identified and pseudonyms allocated to all qualitative information.

### 2.5 Participants

Patients were eligible to participate in the study when they had transitioned from hospital to home, were aged 70 years or older, experienced at least two chronic health conditions and spoke English sufficiently to provide informed consent. Carers were eligible to participate when they were in the role of an unpaid informal carer as nominated by the patient. Carers were eligible when they were aged 18 years or over and spoke English sufficiently to provide informed consent. Patients and carers with cognitive impairment were excluded from the study.

Purposive sampling was used to select, for a semi‐structured interview, up to 20 patients, plus or minus their carers/family, who had recently transitioned from participating wards at the health‐care network to their own home. The purpose of the semi‐structured interviews was to describe care transition trajectories and experiences in depth. From individual interviews, similar codes and categories emerged after 12 interviews. A further eight interviews were conducted with similar findings. Therefore, data saturation was considered to be established after 20 interviews.

### 2.6 Instruments and interview guidelines

Instruments and interview guidelines included a demographic questionnaire for patients, and carers/family, and semi‐structured interview guidelines prompting responses regarding care recipients’ experiences of care transitions, what they valued, and what was missing. Instruments and interview guidelines were developed from a previous literature review,^7^ systematic review,^31^ meta‐synthesis review^36^ and qualitative pilot study.^37^ In addition, we used recommendations by Bate and Robert^34^ to develop the interview guidelines.

### 2.7 Procedure and data collection

Senior nurses on each ward were invited to identify patients who met the selection criteria and introduce the study to these patients. With the patient's permission, the first author then used the Participant Information Form to explain the study. The researcher invited the person to complete a screening and demographic questionnaire to establish their eligibility to participate in the study. The researcher then invited eligible people and, if acceptable, their carer to participate in a face‐to‐face semi‐structured interview of approximately 1‐hour duration at home at least 1 week following discharge. Patients who agreed to be interviewed were invited to nominate their informal carer to participate. Consenting carers were invited to complete the relevant screening and demographic questionnaire prior to commencement of the interview. Following an explanation of the study guided by the Participant Information Form, written consent from patients and carers was obtained just prior to the interview. With permission, the semi‐structured interview was audio‐recorded. The first author conducted the interview and transcribed the data.

### 2.8 Data analysis

Demographic information for patients and carers was entered into Statistical Package for the Social Sciences (SPSS) version 21. Categorical data were analysed for frequencies, and continuous data were analysed using descriptive statistics.^38^, ^39^

Qualitative data were analysed using the inductive data analysis technique of thematic analysis.^40^, ^41^, ^42^ Thematic analysis was guided by the research question and was an iterative process involving the comparing and contrasting of codes and categories within interviews and between interviews to derive themes.^33^, ^43^, ^44^ The authors discussed and interrogated codes, categories and themes to test interpretations of data and support a plausible and coherent interpretation of the data.^42^

## 3 FINDINGS

**Figure 1:**
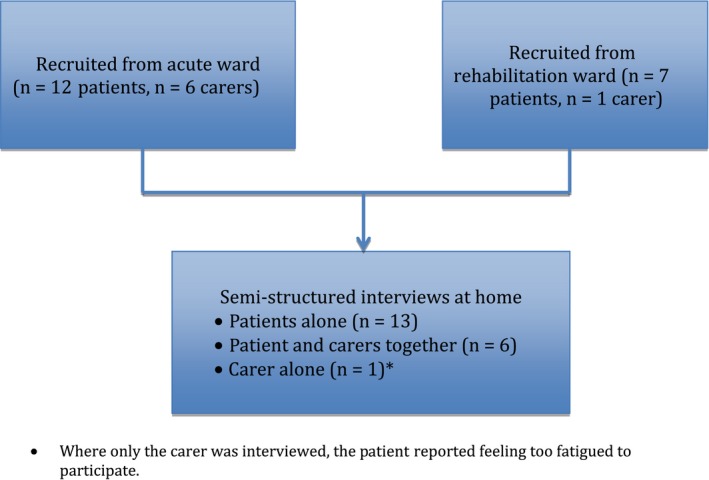
Recruitment location and interview participation

A total of 20 interviews were conducted. Thirteen interviews were with a patient, six interviews were with a patient and carer together, and one interview was with a carer. Details regarding recruitment location and interview participation are presented in Figure 1. The average length of time between the date of hospital discharge and the interview at home was 25 days (range of 8‐88 days). On average, interviews were 37 minutes in length (range of 17‐60 minutes). Participants were aged on average 78.9 years (age range 45‐94 years), and 16 (61.5%) were female. Demographic information is presented in Table 1 for patients and in Table 2 for carers.

**Table 1:** Demographic characteristics of patient participants (n = 19)

Demographic information	Frequency (%)
Country of birth	
Australia	10 (52.6)
Netherlands	3 (15.8)
Other	6 (31.6)
Occupation before retirement	
Clerical	5 (26.3)
Business	3 (15.8)
Nursing	3 (15.8)
Other	8 (42.0)
Receives old age pension	
Yes	16 (84.2)
No	3 (15.8)
Has informal carer	
Yes	19 (100)
No	0 (0.0)
Carers’^a^ relationship with the person	
Spouse	5 (26.3)
Daughter/son	12 (63.0)
Other	2 (10.5)
Lives alone	
Yes	13 (68.4)
No	6 (31.6)

a Not all carers were interviewed.

**Table 2:** Demographic characteristics of carer participants (n = 7)

Demographic information	Frequency (%)
Country of birth	
Australia	5 (71.4)
Other	2 (28.6)
Occupation (current or prior to retirement)	
Home duties	3 (42.9)
Business	2 (28.6)
Personal care attendant	2 (28.6)
Receives old age pension	
Yes	5 (71.4)
No	2 (28.6)
Carer lives with the person	
Yes	5 (71.4)
No	2 (28.6)
Carer lives alone	
Yes	2 (28.6)
No	5 (71.4)

Six main themes were identified that described participants’ transitional care experience.

1. Needing to become independent.
2. Supportive relationships with carers.
3. Caring relationships with health‐care practitioners.
4. Seeking information.
5. Discussing and negotiating the transitional care plan.
6. Learning to self‐care.

### 3.1 Needing to become independent

All patient participants described the need to become independent. All participants reported a range of health problems resulted in their admission to hospital and in their dependence upon health‐care practitioners for care, including falls, infection, weight loss, fatigue, shortness of breath and difficulty walking. All participants noted they were independent adults who had been dependent upon others for care in hospital and continued to require support from carers and community‐based health‐care practitioners at home. Some patients perceived that they were independent adults across their lifespan yet they needed to adjust and adapt to being slower following their hospitalization. Some participants associated being dependent in hospital with being confined and incarcerated. Other participants reported that being at home was freedom. Most patient participants stated that they valued their independence and wanted to return to independence at home as quickly as possible after being in hospital. According to a patient from rehabilitation:

(Participant 16) Coming home [is the most important thing], you are in your own routine. You can do what you like and everything. … I'm independent. I want to do as much as I can.

### 3.2 Supportive relationships with carers

All patient participants emphasized that supportive relationships with spouses, family, friends and neighbours were reassuring for them, assisted them in their recovery at home after hospital discharge and assisted them to become independent with support. All patients described supportive relationships with carers in terms of practical support, including assistance with safety at home, personal care, self‐management and transportation. All participants explained that family, particularly spouses, supported the person in managing activities of daily living, including showering and housekeeping, managing urinary catheters and managing medications.

In addition to practical forms of support, all participants reflected that family and friends provided emotional support and reassurance. According to these participants, being home with the support and encouragement of family and friends assisted them to feel reassured, secure and regain confidence in their independence.

According to all participants, supportive relationships with carers were essential for the person to remain at home and become independent either with or without continuing formal home support after hospital discharge. Two participants commented that family members struggled to provide support due to their own health difficulties and this contributed to re‐admissions to hospital. Most participants agreed that family support was crucial to preventing re‐admission to hospital.

### 3.3 Caring relationships with health practitioners

All participants discussed the importance of caring relationships with health‐care practitioners in becoming independent in their care transition. According to these participants, nurses and medical practitioners who were friendly, helpful, and who explained care interventions and transitional care plans promoted a sense of being cared for as a person, and this supported their confidence at home. According to many participants, nurses in rehabilitation attended to follow‐up phone calls to check how they were managing after discharge home. These participants valued this care because they felt cared for as a person and it supported their sense of confidence in being at home. Most participants reported feeling cared for as a person when they were able to say goodbye at discharge, as stated by a patient from the rehabilitation ward:

(Participant 8) After 10 weeks, the nurses, I almost got to know them personally, all of the nurses and staff. And when I was being wheeled out in the wheel chair to go I passed their room and there were 10‐12 of them having their morning tea and they all stood up and waved [patient became teary].

Some participants described how negative experiences of caring relationships with health practitioners resulted in uncoordinated and ad hoc transitional care. They perceived that when nurses and medical practitioners involved in their care transition did not introduce themselves and the participant did not know the identity of the health‐care practitioner this resulted in an uncoordinated care transition. These participants further perceived lack of continuity of medical practitioners and interactions with multiple medical practitioners who did not introduce themselves, did not explain their medical diagnoses or their continuing treatment, limited participants’ trust in medical practitioners. This was because they did not know who the different medical practitioners were or how they were attempting to assist them. Some participants perceived that medical practitioners did not listen to their accounts of symptoms and made decisions about discharge medications without understanding treatments and medications prescribed by other medical practitioners. According to one carer, nurses and medical practitioners did not listen to the family's concerns about the person during discharge and transitional care over several years despite frequent hospital admissions in acute care. This resulted in difficulty accessing aged care and an assessment from a geriatrician.

Several participants reported their perceptions of uncaring transitional care to home including no follow‐up services in place and intravenous tubing remaining in the person's arm. According to two participants, family and friends were asked to collect the person in the evening following administration of blood products. They noted that this would have resulted in discharge home alone, with no food in the house or home in the care of a spouse with dementia. These carers perceived that this was uncaring, unsafe, and an uncoordinated discharge.

All participants perceived positive caring relationships with health practitioners when the person and carer's needs and wishes were listened to, considered and included in transitional care planning, supported their return to independence at home. According to some participants, when health‐care practitioners did not include participants in discussion about their discharge, or when health‐care practitioners did not share health information with them or listen to them they felt their needs were not considered. These participants perceived that important information was overlooked, including symptoms suggesting a potential urinary tract infection, memory problems and limited carer availability for ongoing support at home.

All participants needed reliable and consistent transitional care to assist them to know what to expect, to plan their day and to feel reassured and confident in their transitional care. Many participants described care continuity with health‐care practitioners was important for their perception of co‐ordinated and consistent transitional care. Participants reported that they believed continuity of care resulted when nurses in hospital and at home provided consistent nursing care, pharmacists provided consistent information and education regarding discharge medications, and follow‐up home visits occurred as planned from all services.

### 3.4 Seeking information

To become independent at home following hospital discharge, most participants sought information about their medical diagnoses and treatments. They wanted to know what changes had been made to their medications and the reason for these changes. When the person was too unwell to seek this information during their acute illness, their carers wanted to know this information on their behalf. Most participants expected medical practitioners to share this information with them during the hospital admission; however, this did not always occur. All participants receiving care from the rehabilitation ward noted they were well informed of their medical diagnoses and changes in their medications. All participants reported that they valued information from the ward pharmacist regarding the nature and purpose of their discharge medications.

According to some participants, information about medical diagnoses, treatments and medications became more problematic when multiple medical practitioners were involved in their care. Some participants considered that they should have asked more questions of the medical staff; however, they did not know what questions to ask.

According to all patient and carer participants, the GP was an essential source of information about medical diagnoses, treatments and changes in medications in hospital. They noted that their GP relied on an accurate and timely discharge summary to explain this information. Many participants valued GPs who made time to explain, clarify information in the discharge summary, and answer their questions about recovery and rehabilitation at home. A carer from the acute ward commented:

(Participant 3) When we saw our GP yesterday, she described in great detail exactly the significance and the severity of a bug in the blood if it was coming from the bladder. So we got more out or our GP in 5 minutes than we got out of the doctors in the hospital in 8 days.

### 3.5 Discussing and negotiating the transitional care plan

Many participants reported discussing and negotiating their transitional care plan with health‐care practitioners and this assisted them in regaining their independence once at home. Participants reported discussing their wishes regarding their care transition including continuing supports and follow‐on care at home. Some participants reported that they wanted to be discharged in the evening so that their family could pick them up without having to take time off work. They valued being able to discuss and negotiate this with medical staff. Several participants declined follow‐on care at home because they considered that they did not require such care. They also valued being able to discuss and negotiate this with medical and nursing staff during their hospital discharge and being reassured that they could contact the hospital if they needed to do so after discharge.

Several carers perceived that they negotiated the time and date of hospital discharge and the location for the care transition. Two carers declined hospital discharge on behalf of the person because they perceived that it was not safe to send the person home late at night. One patient considered that a family member negotiated inpatient rehabilitation for him instead of inpatient respite and he was grateful for his niece's support:

(Participant 6) When they'd agreed that rehabilitation was the thing and then this other doctor comes wandering in and starts saying “No, No, No, that's not going to happen.” And starts talking about respite care. I'm thinking that doesn't sound awfully helpful. That was when my niece took control of the situation.

### 3.6 Learning to self‐care

All participants perceived that they engaged in learning to self‐care to become independent. They explained that they learned to self‐care in relation to complex medication regimes, colostomies and urinary catheters, challenging symptoms and conducting and interpreting blood glucose monitoring.

Some participants reported hospital and community‐based health‐care practitioners provided formal education regarding self‐care of relevant long‐term health issues. These participants noted that education in the inpatient setting was focussed on management of technical aspects of self‐care. All participants who had received care in the rehabilitation ward also commented that they received education focussed on continuing rehabilitation to strengthen mobility and function. All participants valued medication education from the ward and community pharmacists including education regarding dose administration containers, explanation of discharge medication regimes and consideration of unwanted side‐effects. Many participants explained they consulted with their GPs about adapting care routines to control symptoms, and about the reasons for changes in their medications. Some participants living with diabetes noted that their GP supported their learning about continued monitoring and interpretation of blood glucose levels in relation to diet and activity. They reported collaborating with their GP to learn how to make the best decisions for their self‐care in relation to living with their chronic disease and living a good quality of life. Community‐based nurses were considered to provide education about long‐term care such as leg care, self‐care of diabetes, self‐care of urinary catheters and colostomy management.

Many participants explained that nurses on the acute medical ward routinely provided written patient discharge information, which was helpful to understanding their self‐care needs. Other participants noted that this written information was not helpful as it was very general and not specific. Some participants commented that education from allied health professionals regarding the use of aids and equipment in the home was not helpful because use of this equipment was self‐evident.

Some participants described closely watching nurses in hospital give insulin injections, check their blood glucose levels, manage their urinary catheters including changing urine collection bags and manage their stomas. In this way, they considered that they learned how to manage these more technical aspects of their self‐care. These participants further explained that observing nurses conducting their care also provided opportunity to ask questions supporting self‐care learning.

Many participants reported self‐care also involved discovering resources, such as services and supports, which were available to them in the community. These participants explained that they discovered available resources when they engaged in questioning and discussion with health‐care practitioners, and with other people including carers, family and friends about what services were available at home.

**Figure 2:**
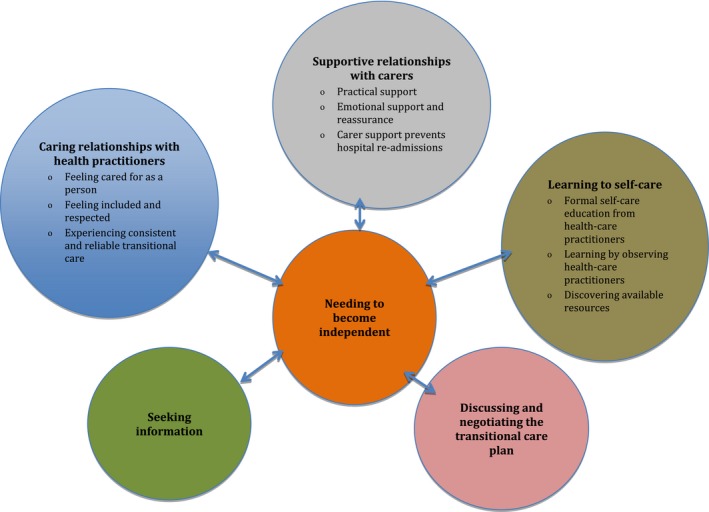
Patients’ and carers’ perspectives of their transitional care experiences

Themes and subthemes reflecting participants’ perspectives in relation to their transitional care experiences are summarized in Figure 2.

There were no substantial differences in the findings identified among those interviewed with and without their carer present.

## 4 DISCUSSION

Findings from the 20 semi‐structured interviews with patients and carers reflect the need for care recipients to become independent in their care transition. Demographic data suggest that participants were a largely Anglo‐Australian group, had carer support and were of middle‐income socio‐economic status.

The desire to be independent emerged as the problem that required a solution in care transitions. This theme suggests that regaining independence is an important continuing care need and goal of recovery and supports and expands on findings from other studies. In a meta‐synthesis review, user experience of transitional care was a social process of negotiation and navigation of independence for older people and carers.^36^ Other research has also found that independence is important to older people living in the community both with and without support.^45^, ^46^, ^47^ The current finding contributes to our understanding that ideal transitional care interventions should focus on patients’ desire and need to regain independence as well as on communication between practitioners about the discharge assessment and plan of care; preparation of the person and carer for the care transition; reconciliation of medications at transition; a plan for follow‐up care; and patient education about self‐management.^1^, ^15^

In this study, participants’ solutions are understood as social processes directed at needing to become independent. In being dependent on carers and on health‐care practitioners during care transitions, patients prioritised quality caring relationships: supportive relationships with carers, and caring relationships with health‐care practitioners. However, patients and carers could also be active self‐managers as reflected in the themes: seeking information, discussing and negotiating the transitional care plan, and learning to self‐care. These solution‐oriented themes mirror other research findings.^31^, ^36^ Support from carers and health‐care practitioners and self‐management were emphasized in previous studies about the effectiveness of transitional care interventions with good patient satisfaction ratings and reductions in readmission rates.^18^, ^19^, ^21^, ^25^, ^48^ The current study findings also expand on previous research by highlighting quality care transitions as inherently social. Findings emphasize care recipients as active users of transitional care interventions. Social processes supporting the strengths and capacities of patients and their carers should be emphasized in future transitional care initiatives.

Findings from the semi‐structured interviews provide important insights into what it is like to be a patient and carer living with chronic health difficulties and needing to find strategies and solutions to become as independent as possible in transitional care. However, transitional care is complicated by the barriers of health service fragmentation and increased demand on health‐care services due to the ageing of the population.^9^, ^10^, ^11^ Consequently, in care transitions, health‐care practitioners and services focus on rapid throughput and efficiencies for health‐care services.^31^ From the perspective of user experience, this presents a potential dilemma in transitional care. Future interventions should emphasize strategies to support negotiation of transitional care needs and supports, and assist care recipients to overcome these barriers. These interventions should comprise example questions about the discharge plan that care recipients and carers can ask health‐care practitioners. Statements explicitly noting that care recipients and carers are permitted to ask questions about hospital discharge should be built into these care strategies.

As transitional care is integral to efficient and safe health‐care systems, it also needs to be built into and across acute, subacute and community‐based care systems. Findings from the current study add knowledge about important social processes to include in future research and practice initiatives for use by health‐care practitioners in general roles with limited aged care or community care expertise.

### 4.1 Study limitations

As our sample was limited to patients with carers, it is difficult to extrapolate the findings to those transitioning through the health system with little or minimal carer or family support. Patients and carers from low socio‐economic areas, with limited carer support and patients from culturally and linguistically diverse communities, did not participate in the current study. Their perspectives may provide important insights for intervention development to meet their particular needs and requirements. Although carers were included, their involvement was mainly in interviews with patients. This may have limited their sharing of their own care needs.

## 5 CONCLUSION

Despite these limitations, increasingly, patients and carers are expected to be involved in decisions about their care including care transitions.^5^ Governments, policymakers and health‐care services are all expecting patients and carers to be more involved in their care and to take greater responsibility for their continuing care needs at home.^9^ Future care interventions should be designed to fit with these imperatives.

Findings suggest that some patients and carers in public health‐care systems characterized by acute, subacute and community‐based care have distinct user needs in care transitions and use a range of social processes as solutions. This is an important and potentially complicating factor because it may not be the focus of health‐care practitioners. New approaches and strategies, such as communication aids and assessment tools with specific questions that guide care recipients towards independence following discharge, are necessary in transitional care to support questioning, discussion and negotiation between patients, carers and health‐care practitioners. All health‐care practitioners in acute, post‐acute and community‐based care should participate in continuing education that emphasizes the care recipient's role in negotiating their discharge with practitioners. New approaches and strategies could contribute to improved user experiences in the challenging area of transitional care for older people living with chronic health problems and their carers.

## CONFLICT OF INTEREST

The authors declare no conflict of interest in this study.
